# Assessment of Vegetative Growth and Genetic Integrity of *Vanilla planifolia* Regenerants after Cryopreservation

**DOI:** 10.3390/plants11131630

**Published:** 2022-06-21

**Authors:** María Teresa González-Arnao, Carlos A. Cruz-Cruz, Fabiola Hernández-Ramírez, Jorge A. Alejandre-Rosas, Ana Constanza Hernández-Romero

**Affiliations:** Laboratorio de Biotecnología y Criobiología Vegetal, Facultad de Ciencias Químicas, Universidad Veracruzana, Orizaba, Veracruz 94340, Mexico; calcruz@uv.mx (C.A.C.-C.); fabiolhernandez@uv.mx (F.H.-R.); jalejandre@uv.mx (J.A.A.-R.); a.constanza_hdz@hotmail.com (A.C.H.-R.)

**Keywords:** vitrification-based techniques, cryo-derived plants, greenhouse, ISSR molecular markers

## Abstract

*Vanilla planifolia* Jacks. ex Andrews is the vanilla species with the most commercial and greatest economic importance. It has been used as a case study in different cryopreservation studies that involve three vitrification-based approaches: droplet-vitrification (D-V), V-cryoplate (V-Cp) and D-cryoplate (D-Cp). The aim of this study was to compare the impact of these cryogenic techniques on vegetative growth (survival, stem length and leaf number) between cryo-derived plants and in vitro-derived controls during 12 months of greenhouse growth. Genetic stability was also assessed using the inter-simple sequence repeat (ISSR) markers. There were no significant differences found in the survival and stem lengths of the in vitro-derived regenerants and cryo-derived plants. A significant increase in the number of leaves was only detected in cryo-derived plants when using the V-Cp method. The electrophoretic profiles, based on seven ISSR primers, detected low variability: 81 total bands and 27% polymorphism. This is the first report on the assessment of vegetative growth and genetic integrity in cryo-derived *V. planifolia* plants recovered under greenhouse conditions. Of the three cryogenic approaches, D-Cp appears to yield *V. planifolia* regenerants plants with more vigorous vegetative growth and a lower level of polymorphism. Future research should focus on the reproductive growth of vanilla regenerants.

## 1. Introduction

Among the cultivated vanilla species, *Vanilla planifolia* Jacks. ex Andrews is the most important worldwide, as it is the natural source of the famous multi-industrial vanilla extract [1].

Vanilla is a monopodial and climbing orchid characterized by a single robust stem with oblong, thickened waxy leaves. Aerial roots and flower stems emerge from nodes found along the stem next to the leaves [2]. This emblematic orchid is native to the tropical areas of Mexico and is the subject of legends, customs and traditions in the State of Veracruz [3,4].

Different biotechnological techniques, such as tissue culture [5,6,7], temporary immersion bioreactors [8,9], genetic transformation [10] and cryopreservation [11,12] have been studied for the in vitro propagation and conservation of *V. planifolia* germplasm. For instance, the cryopreservation of vanilla shoot-tips was reported for the first time in 2009 [12] and since then, different cryo-biotechnological investigations have been carried out to optimize and refine a robust and reproducible protocol [11].

Vitrification-based procedures, such as droplet-vitrification (D-V) [12], V-cryoplate (V-Cp) [1] and D-cryoplate (D-Cp) [11] led to differential survival and regeneration percentages following the cryopreservation of *V. planifolia* shoot-tips. Using D-V, the main problem was associated with oxidative damage [12]. Following the V-Cp and D-Cp methods, the difficulties were associated with the transient de-differentiation of tissues after their exposure to liquid nitrogen [1,11]. However, despite some detrimental effects on survival and recovery, as mentioned above, all techniques allowed regrowth in various percentages of cryopreserved vanilla explants. In all cases, the regenerated plantlets were first recovered in vitro and then successfully transferred to greenhouse conditions [13].

No matter how optimized a cryogenic protocol is, the impact on the genetic stability of viable regenerants should always be verified. There are several useful markers to assess the genetic integrity of in vitro cultured and cryo-derived plants. One of the most reliable and simplest techniques is the inter-microsatellite sequencing through the ISSR (inter-simple sequence repeat) markers [14]. These molecular markers have been successfully used in different plant species [15,16,17], and more specifically, for various in vitro studies in *V. planifolia* [7,18].

The vegetative growth studies [19], which can include the assessment of morphological features [15] and/or field performance [20], represent a useful approach for the assessment of cryo-derived plants. Unlike the molecular markers, these complementary studies also accommodate for the plant growth responses to environmental conditions [21].

The aim of this study was to evaluate the vegetative growth (survival, plant height and leaf number) during a greenhouse culture of regenerated plants after the cryopreservation of *V. planifolia* shoot-tips. Three cryogenic techniques were compared: D-V, V-Cp and D-Cp. The genetic stability of the cryo-derived and in vitro-derived plants was also assessed using ISSR markers.

## 2. Results

### 2.1. Cryopreservation of Shoot Tips from In Vitro-Grown Plants

The results after the cryopreservation of vanilla shoot-tips were very similar to that previously reported by Hernández-Ramírez et al. [11] and González-Arnao et al. [1], using the same three procedures. The post-cryopreservation recovery was quite variable, except when the D-Cp protocol was used. The highest regeneration rate (33%) was obtained following the D-Cp method, with only a 10% regeneration after the D-V and V-Cp protocols.

### 2.2. Vegetative Growth in Greenhouse Culture

No significant differences (*p* ≤ 0.05) were found in the survival (%) between the in vitro controls and cryo-derived plants after acclimatization and the same culture period in the greenhouse (Table 1). The average survival rate remained relatively stable during plant growth, ranging from 72% (at 3 months) to 65.5 % (at 12 months). The greatest decline from 100% to 72% occurred during the first three months of acclimatization, which could be attributed to the adaptation period of plants to the ex vitro culture.

### 2.3. Stem Length of Acclimatized In Vitro and Cryo-Derived Plants

When compared at the same culture periods, the stem lengths (cm) of the in vitro controls and cryo-derived plants were not significantly different regardless of the technique used (Table 2). The initial stem length of the in vitro and cryo-derived plants was 12.08 cm on average. It increased on average to 2.24, 4.52, 7.01 and 9.74 cm after 3, 6, 9 and 12 months of growth in the greenhouse, respectively. The elongation of the recovered plants after using the D-V and D-Cp techniques did not show significant differences from nine months of growth onwards (Table 2, indications in lowercase letters).

### 2.4. Leaves Number of Acclimatized In Vitro and Cryo-Derived Plants

A significantly higher number of leaves were recorded in cryo-derived plants when the V-Cp method was used (Table 3). The highest production was detected both in the same culture period and throughout the 12 months of growth. The total number of leaves increased on average to 1.25, 2.56, 3.99 and 5.47 units after 3, 6, 9 and 12 months of growth in the greenhouse, respectively.

**Figure 1:**
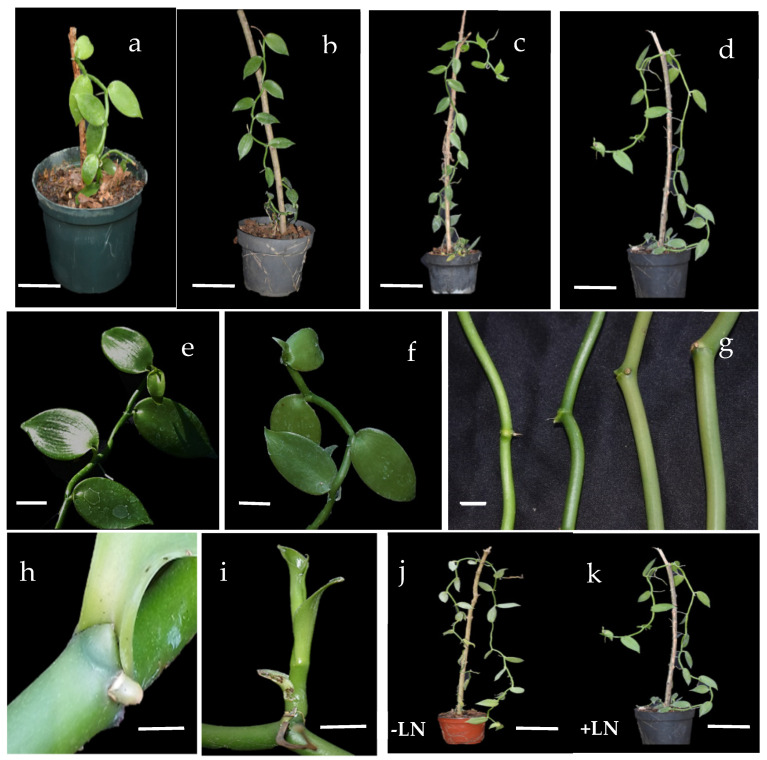
Vegetative growth of greenhouse-cultured plants of *V. planifolia*. Cryo-derived regenerants produced using the D-Cp method and grown for (**a**) 3 months (bar 5 cm), (**b**) 6 months (bar 5 cm), (**c**) 9 months (bar 10 cm), and (**d**) 12 months (bar 10 cm) in the greenhouse. Leaves (**e**) from in vitro-control (bar 2 cm) and (**f**) from D-Cp method (bar 2 cm) regenerants. Vanilla stems (**g**) from in vitro-control, D-V, V-Cp, and D-Cp regenerants. (**h**) Axillary bud (bar 1 cm), (**i**) newly developed shoot from D-Cp method regenerants (bar 1 cm). (**j**) Plant from in vitro-control (not subjected to cryopreservation, -LN) (bar 10 cm) and (**k**) cryo-derived plants (+LN) using D-Cp method (bar 10 cm)—both grown in the greenhouse for 12 months.

Similar vegetative growth was observed in the cryo-derived plants and the in vitro controls after 12 months of greenhouse culture. Likewise, the leaf shape and plant color were also visually similar. However, the stems from cryo-derived (D-Cp > V-Cp > D-V) plants were slightly thicker (~up to 0.5 cm) compared to the in vitro controls (Figure 1).

### 2.5. Assessment of Genetic Integrity of Greenhouse-Grown Plants by ISSR Analysis

The analysis of the electrophoretic profiles, according to the seven ISSR primers (T05, T06, C07, UBC-823, UBC-836 and UBC-840), revealed a total of 81 bands (286 amplicons), ranging from 320 to 4000 bp. Of these, 59 bands were monomorphic and 22 were polymorphic, which represented 27% of total polymorphism.

**Figure 2:**
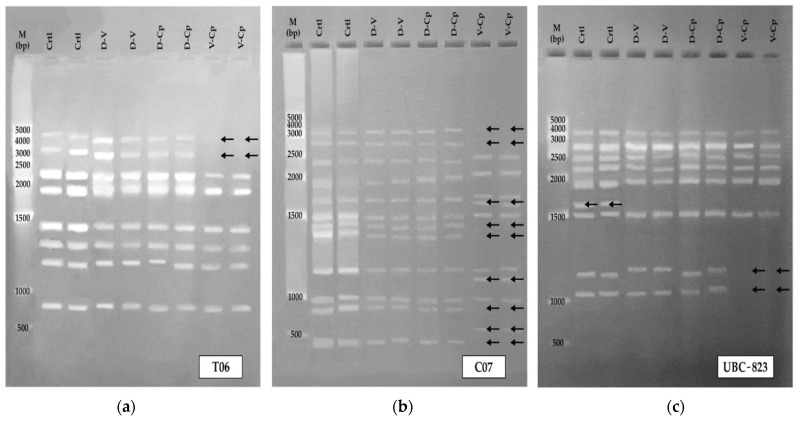
Electrophoretic patterns using ISSR primers (**a**) T06, (**b**) C07 and (**c**) UBC-823 of in vitro-derived controls and cryo-derived *Vanilla planifolia* plants during greenhouse culture. Arrows indicate presence or absence of polymorphic bands. M: molecular weight marker, Ctrl: control, D-V: droplet-vitrification, V-Cp: V-cryoplate, D-Cp: D-cryoplate.

Of the studied ISSR primers, five (T06, C07, UBC-823, UBC-840, UBC-848) produced well-defined polymorphic bands. The main differences in banding patterns were observed in the cryo-derived plants when using the V-Cp method (by T06, C07 and UBC-823 primers), and in the in vitro controls (by UBC-823, UBC-840, UBC-848 primers). Figure 2a–c shows the selected examples of different electrophoretic profiles.

**Figure 3:**
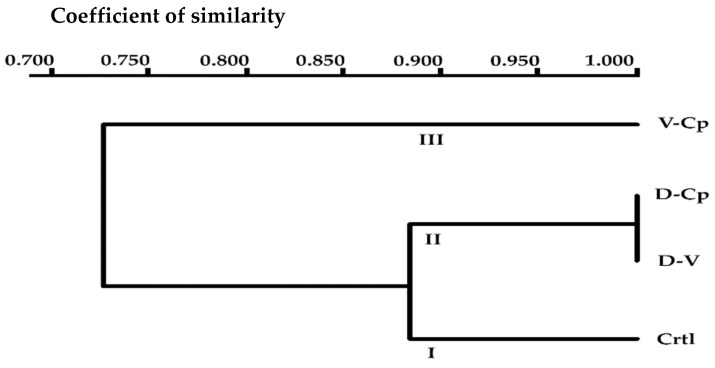
Genetic similarity dendrogram based on Jaccard’s coefficient. Neighbor-joining analysis after six months of greenhouse culture of in vitro controls (Ctrl) and of cryo-derived regenerants produced using three vitrification-based techniques.: D-V: droplet-vitrification, V-Cp: V-cryoplate, D-Cp: D-cryoplate. The values of coefficient of similarity represent: 1 similarity and 0 divergence.

The summary of the analysis with the ISSR primers is presented in Table 4. A UPGMA dendrogram, based on the similarity coefficient of Jaccard, is represented in Figure 3. According to the cluster analyses, the dendrogram showed the formation of three groups. The first group was formed only by the in vitro-derived plants (non-cryopreserved control). The second group, with an 88% similarity, was composed of the cryo-derived plants produced using the D-V and D-Cp procedures, and the third group, with a 73% similarity, was composed solely of the cryo-derived plants produced using the V-Cp method. Groups II and III, both composed of cryo-derived plants, had the least (17%) genetic distance between them, showing an 83% similarity.

## 3. Discussion

Different studies have shown that cryopreservation results in little to no changes in the genetic stability of recovered plants [20,21,22]. However, the development of a reliable cryopreservation protocol requires the optimization of several factors/steps that involve cells undergoing ultrastructural, morphological, and molecular changes [23]. Cryogenic processes impose stressful conditions that could affect the genetic integrity and vegetative growth of regenerated plants [20]. For this reason, any cryopreservation procedure must be verified and validated before its routine use.

In general, the results obtained in the present work indicated no significant differences in the vegetative growth between the cryo-derived and the in vitro-derived control plants during the greenhouse culture for 12 months. Based on the characteristics compared (the stem length and number of leaves), a significant increase in the number of leaves was only detected in the cryo-derived plants when using the V-Cp method (Table 3). In light of this, it is expected that the greater number of leaves could result in the increased generation of new shoots and a faster lateral development, since the axillary buds of *V. planifolia* are anatomically located in the upper part of each leaf. However, after 12 months of culture, only about one new shoot per plant was detected, regardless of whether the plants were from the cryo-derived or in vitro controls (data not shown). This allowed us to visually detect that this quantitative characteristic did not induce a greater lateral growth, since apical dominance prevails and, consequently, the development of the plants is given by the elongation of the main stems, among which there were no significant differences detected. Despite the lengths being not statistically different, the cryo-derived plants had slightly thicker stems than the in vitro controls (Figure 1g), demonstrating that cryo-derived plants grew more vigorously after the same culture time.

The increase in the number of leaves detected during the greenhouse culture in cryo-derived plants from the V-Cp protocol could be associated with a ‘hormetic effect’. According to Calabrense [24], the hormetic effect is characterized as a beneficial or stimulating development after losing doses of certain stressors that the biological material is able to overcome. During the in vitro culture, this stimulus in leaf production could very well go undetected; however, it was evident under the greenhouse culture conditions, possibly because the plants become autotrophic. The increase in the production of leaves during the vanilla culture could result in improved growth, particularly if it interrupts apical dominance in strong and well-developed plants.

Different responses have been found after comparing the vegetative growth of the cryo-derived plants of various species. Bi et al. [15] found that the vegetative growth of cryo-derived *Chrysanthemum morifolium* plants was less vigorous in the early stage of greenhouse culture. By contrast, Castillo et al. [21] determined that after 1 year of growth in the field, all cryo-derived *Rubus* plants were more vigorous than the greenhouse mother plants. On the other hand, a field performance comparison of the cryo-derived, micropropagated, and field sucker-propagated *Musa* plants showed similar vegetative and reproductive growth among the three plant sources [25].

Therefore, data regarding the vegetative growth of the cryo-derived *V. planifolia* plants for 12 months in a greenhouse is in agreement with the observations of similar studies in several species. The next step for the assessment of the vanilla regenerants will, therefore, be to compare the reproductive growth between the control and cryo-derived plants.

Genetic stability has also been assessed in the cryo-derived regenerants of different plant species [15,21,26]. The ISSR markers have been frequently used to evaluate the effect after the cryopreservation of shoot tips following various vitrification-based techniques, such as droplet-vitrification [15,16], encapsulation–dehydration [16,27], encapsulation-vitrification [28] and vitrification [29].

Of the ISSR primers tested in this study, five revealed a 27% total polymorphism in the regenerants derived from the cryopreserved vanilla shoot-tips (Table 4). The V-Cp method showed a higher polymorphic rate than the D-V and D-Cp procedures with respect to the in vitro controls and among the three compared cryo-techniques. These results indicated that the genetic stability may vary depending on the cryogenic procedure. The main causes have been attributed to the sub-optimal cryoprotective conditions and in vitro culture before and after liquid nitrogen immersion [30].

Martín and González-Benito [31] detected differences in the genetic stability of *Chrysanthemum* cryopreserved using the vitrification and encapsulation–dehydration methods. They found that variations occurred at low frequency (1 regenerant out of 25 showed a different band pattern) and followed the encapsulation–dehydration method only.

Until now, most of the molecular analyses performed on *V. planifolia* have focused on the impact of the in vitro culture techniques. Some studies have detected 71.66% polymorphism in plantlets regenerated by indirect organogenesis [5]; others, more than 15% polymorphism after six-subculture cycles during micropropagation [7], and Bautista-Aguilar et al. [18] found low polymorphic percentages (2%) when using six ISSR primers to evaluate the genetic stability of *V. planifolia* and *V. insignis* shoots subjected to in vitro conservation by minimal growth.

By using the ISSR markers, genetic stability was also assessed in the in vitro regenerants of *Vanilla planifolia* that were propagated for 6 months after the recovery of each successive step of a droplet-vitrification protocol [32]. The cluster analysis, based on Nei’s genetic distance, revealed very close similarity coefficients (0.816 and 0.819) for shoots regenerated before (PVS2-treated, 12.7% polymorphism) and after liquid nitrogen immersion (7.8% polymorphism), respectively. These preliminary results apparently indicated that immersion in liquid nitrogen did not cause additional genetic variations, while the treatment with PVS2 induced the greatest variation [32].

A comparison of the effects of the D-V, V-Cp and D-Cp techniques on the genetic stability of the *V. planifolia* regenerants during the in vitro (González-Arnao et al., unpublished results) and ex vitro (greenhouse) cultures detected a similar polymorphism using the same (T06) ISSR primer in both studies: D-V (22% for the in vitro and 25% for the ex vitro plants), V-Cp (25% for the in vitro and 33% for the ex vitro plants) and D-Cp (25% for the in vitro and ex vitro plants). Therefore, it is possible to speculate that, after acclimatization, no additional variations to those previously observed under the in vitro conditions were generated.

In summary, to the best of our knowledge, this is the first report on the evaluation of vegetative growth and genetic integrity in cryo-derived *V. planifolia* plants grown under greenhouse conditions and the comparison of the three cryogenic approaches focused on here. Based on the overall results, D-Cp appears to be the most recommendable cryopreservation method for *V. planifolia*. According to Hernández-Ramírez et al. [11], this procedure is the most optimized and reproducible for shoot tips of this vanilla species. As observed here, the greenhouse-grown regenerants produced using this technique showed more vigorous vegetative growth and low levels of polymorphism (average 27%). In comparison to the D-V and V-Cp methods, the D-Cp procedure does not require the use of toxic substances at high concentrations, such as dimethyl sulfoxide (DMSO), polyethylene glycol and glycerol, which are components of the vitrification solution, PVS2 [33], and which may induce a genetic variation during a cryopreservation process [30].

## 4. Materials and Methods

### 4.1. Cryopreservation of Shoot Tips from In Vitro-Grown Plants

The apical shoot tips, 3–5 mm in length, were dissected under sterile conditions from the in vitro-multiplied *Vanilla planifolia* Jacks. ex Andrews plants that were subcultured every 12 weeks on an MS [34] semisolid medium supplemented with 1 mg L^−1^ 6-Benzyl amino purine (BAP), 0.5 mg L^−1^ Indole-3-butyric acid (IBA), 20 g L^−1^ sucrose and 7 g L^−1^ agar (Sigma–Aldrich Ltd. Co., St. Louis, Missouri, (MO), USA). The pH of the culture medium was adjusted to 5.7 ± 0.05 and then sterilized in an autoclave at 121 °C with 1.3 kg cm^−2^ of pressure for 15 min. Donor plants were maintained at 24 ± 2 °C under a 16 h light/8 h dark photoperiod with a light intensity of 36 μmol m^−2^ s^−1^ [12].

The dissected shoot tips that were used for the cryopreservation experiments were first preconditioned on a basal-MS-semisolid medium, supplemented with 0.15 M trehalose for 1d, and maintained under the same temperature and photoperiod conditions as donor plants [11]. Afterward, the shoot tips were subjected to three cryogenic procedures: D-V, V-Cp and D-Cp.

Following D-V, the preconditioned samples were loaded with a solution containing 0.4 M sucrose + 2 M glycerol for 30 min and were then exposed to the vitrification solution, PVS2 (30% (*w*/*v*) glycerol, 15% (*w*/*v*) ethylene glycol, 15% (*w*/*v*) DMSO and 13.7% (*w*/*v*) sucrose) [33] for 30 min at room temperature (~25 °C). Afterward, the shoot tips were transferred to PVS2 droplets (of about 15 μL) that were placed on aluminum foil strips (25 mm length × 6 mm width) and were rapidly immersed in liquid nitrogen (−196 °C).

Following V-Cp and D-Cp, the preconditioned shoot tips were first attached with calcium alginate to the surface of aluminum cryoplates (37 mm length × 7 mm width and 0.5 mm thickness) [35]. For encapsulation, tissues were transferred onto a droplet (about 50 μL) of sodium alginate (2%, SIGMA, low viscosity) calcium-free solution, and then, a calcium chloride (0.1 M) solution (75 μL) was gently poured into the borders and over the droplet of alginate to provoke the polymerization for 15 min at room temperature [1]. After the polymerization, the encapsulated samples were treated with loading solutions containing 0.4 M sucrose + 2 M glycerol for 30 min at room temperature. Using the V-Cp procedure, the encapsulated and loaded samples were exposed to PVS2 for 30 min at ~25 °C before being ultra-rapidly immersed in liquid nitrogen. Using the D-Cp method, the encapsulated and loaded samples were desiccated for 180 min in a horizontal laminar flow cabinet at room temperature (~25 °C) and 70–80% RH. After desiccation, the cryoplates were immersed directly in liquid nitrogen.

Regardless of the cryopreservation procedure used, after 1 h of storage at −196 °C, a rewarming of the samples was performed rapidly by plunging the aluminum foils and/or cryoplates into an unloading solution containing 1.2 M sucrose for 15 min. A recovery culture of shoot tips after warming was carried out in a semisolid-MS-multiplication medium in dark conditions for about 4 months until any kind of recovery was detected.

As previously described, the MS-multiplication medium was supplemented with 1 mg L^−1^ 6-Benzyl amino purine (BAP), 0.5 mg L^−1^ Indole-3-butyric acid (IBA), 20 g L^−1^ sucrose and 7 g L^−1^ agar (Sigma–Aldrich Ltd. Co., St. Louis, Missouri, MO, USA). The pH of the culture medium was adjusted to 5.7±0.05 and then sterilized in an autoclave at 121 °C with 1.3 kg cm^−2^ of pressure for 15 min. Two subcultures were performed in 60-day periods prior to rooting in the MS-semisolid medium without growth regulators. The cultures were exposed to 24 ± 2 °C under a 16 h light/8 h dark photoperiod with a light intensity of 36 μmol m^−^^2^ s^−^^1^.

Afterward, all regenerated plantlets derived from the cryopreserved shoot tips were grown in glass tubes using the basal-MS medium to promote the elongation of the aerial part until reaching approximately 10–12 cm in length and favoring the spontaneous formation of well-developed roots [11].

Regenerated plants derived from the non-cryopreserved shoot tips and subjected to the same in vitro culture conditions were used as the in vitro controls for assessing the vegetative growth and genetic integrity of *V. planifolia* plants during greenhouse culturing.

### 4.2. Acclimatization and Vegetative Growth under Greenhouse Conditions

The ex vitro culture experiments were carried out in the greenhouses belonging to the Enterprise of Ornamental Tropical Crops of Veracruz in Cordoba city, Veracruz. The in vitro and cryo-derived 8-week-old plants were acclimatized. The in vitro and cryo-derived plants 8-week-old were acclimatized.

#### 4.2.1. Acclimatization

The culture medium was gently removed from the roots of tissue-culture plants with abundant sterile water, and 1 g L^−1^ fosetyl-Al (aluminum triso-ethyl-phosphonate 80% *w*/*w*) was applied at the base of the roots. The plants were transplanted into plastic pots (11 height × 14 width) containing 250 g of sterilized 387 tezontle (small red basaltic rocks). The pots were covered with a transparent plastic cap and progressively opened every 4 d for 30 d before being completely removed [11]. Afterward, all pots were transferred to a greenhouse with 70% shade, covered by a white plastic caliber 270, at 80% to 95% relative humidity and a temperature of around 30 °C.

After 30 d, the plants were grown in a substrate composed of 50% tezontle and 50% pine bark. Each plant was placed in the substrate 1 cm deep, taking care to cover the base of the stem and the longest roots to avoid dehydration. The aerial roots were left exposed, and a pinewood stake was placed as a supporting tutor. The plants were watered twice a week with running water and fertilized using 1 g L^−1^ Triple 20^®^ (20% nitrogen, 20% phosphorus and 20% potassium). All the greenhouse-grown plants (the in vitro controls and cryo-derived) were carefully monitored to assess their survival (%) and vegetative growth during the 12 months of culture.

#### 4.2.2. Vegetative Growth

The vegetative growth of the in vitro (non-cryopreserved controls) and cryo-derived plants (regenerants recovered after using the three cryopreservation protocols described) was expressed by the increase in stem length (measured from the base of the stem to the upper end of the apical meristem, adding 1 cm, which corresponds to what is sown in the substrate), and by the increase in the number of new leaves formed after 3, 6, 9 and 12 months. The initial stem length, of both the in vitro controls and cryo-derived plants, was 12.08 cm on average. The initial number of leaves, of both the in vitro controls and cryo-derived plants, was 5.88 on average. The survival (%) of the plants was recorded after the same growth periods were referred to as the initial number of planting plants and the number of surviving plants across the culture time. The survival (%) of the plants was recorded after the same growth periods by comparing the initial number of planting plants with the number of surviving plants across the culture time.

### 4.3. Experimental Design and Statistical Analysis during Greenhouse Culture

Survival was analysed by evaluating 50 plants grouped into 5 replicates of 10 samples, randomly selected for both the in vitro controls and cryo-derived plants per each cryopreservation technique and culture period (3, 6, 9 and 12 months) in the greenhouse. The analysis of the stem lengths and the number of formed leaves was performed in triplicate, considering a total of 30 randomly selected plants for both the in vitro controls and cryo-derived plants. All data were analysed with the Minitab^®^ 17 statistical software, State College, Pennsylvania, USA through an analysis of variance (ANOVA) and the Tukey’s test with a significance level of *p* ≤ 0.05.

**Figure 4:**
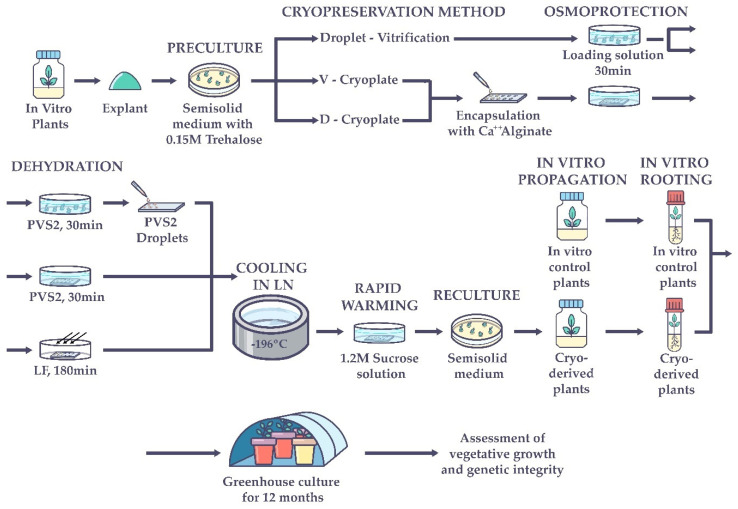
Graphic representation of the experimental work for assessment of vegetative growth and the genetic integrity of *Vanilla planifolia* regenerants after cryopreservation. PVS2: Plant vitrification solution 2. LF: Laminar flow. LN: Liquid nitrogen.

A general illustration of the performed studies in this work is represented in Figure 4.

### 4.4. Assessment of Genetic Integrity of Greenhouse-Grown Plants by ISSR Markers

The leaf samples (0.5 g) from ten randomly selected in vitro controls and cryo-derived plants after six months of culture in a greenhouse were used for DNA extraction. The extraction was performed according to a modified Doyle and Doyle method [36] and carried out in duplicate for each sample. The genomic DNA was quantified by spectrophotometry at an OD ratio of 260/280, and the integrity was verified in 1% (*w*/*v*) agarose gel stained with 2.53 µM ethidium bromide (Sigma–Aldrich, Co., Ltd., St. Louis, MO, USA) and visualized in a UV light using a GelDoc-It photo-documentation system. The ISSR analysis was conducted using seven ISSR primers (Sigma-Aldrich^®^), as presented in Table 5.

The PCR was performed in a 25-μL reaction solution containing: Crystal Buffer (2.5 μL, 10 mM Tris–HCl and 50 mM KCl) (1×), DNTP’s (0.5 μL, 0.2 mM), Taq Polymerase (0.2 μL, 1U/μL), primer (1.0 μL, 0.2 μM) and 1.0 μL of DNA (20 ng). The PCR products were amplified using a MaxyGene^TM^ thermocycler, Axygen Scientific, Union City, CA, USA, under the following program: 4 min at 94 °C, followed by 35 cycles, each comprising 50 s at 94 °C for denaturation, 45 s at 49–56 °C for annealing (depending on the annealing temperature for each primer), 90 s at 72 °C and a final extension of 10 min at 72 °C.

The PCR products were electrophoretically separated on 1.8% agarose gels. The gel was prepared with 1× TBE buffer and stained with ethidium bromide (10 mg mL^−1^). A marker of 500 to 5000 bp was used to visualize the size of the bands. Electrophoresis was performed at 90 V for the first 20 min, followed by 100 V for 1 h, and the gel was visualized in a transilluminator with UV light.

All reactions for each ISSR primer were repeated twice to establish a binary base, scoring 1 for presence and 0 for absence. The total number of bands, the numbers of monomorphic and polymorphic loci, as well as the percentages of polymorphism were calculated. The genetic distances were analysed using the Past v 3.04 program [37] and they were represented as a dendrogram using the unweighted pair-group method with arithmetic mean (UPGMA). The cluster analysis was calculated based on the Jaccard distances.

## 5. Conclusions

The present study focuses on evaluating the vegetative growth and genetic integrity of *V. planifolia* plants recovered after cryopreservation using three vitrification-based methods: droplet-vitrification (D-V), V-cryoplate (V-Cp) and D-cryoplate (D-Cp).

After systematically monitoring plant growth for 12 months in a greenhouse, there were no significant differences found in the general vegetative growth between the cryo-derived plants and the in vitro controls (non-cryopreserved plants). A significant increase in the number of leaves was only detected in the cryo-derived plants when using the V-Cp method.

The genetic integrity assessments revealed 27% total polymorphism using seven ISSR primers. The V-cryoplate procedure resulted in a higher polymorphic rate compared with the D-V and D-Cp techniques and the in vitro-derived control. However, the generally low level of variation suggests that there were no changes in the genetic fidelity of cryo-derived plants.

Currently, this is the most advanced work on the vegetative growth and genetic integrity assessment of cryo-derived regenerants of *V. planifolia*. Future studies will focus on the impact of these techniques on vanilla reproductive growth.

## Figures and Tables

**Table 1:** Comparison of survival between in vitro controls and cryo-derived plants of *Vanilla planifolia* during greenhouse culture.

	Greenhouse Culture Time (Months)			
	3	6	9	12
Method		Survival (% ± SE) *		
In vitro control	68 ± 0.19 Aa	68 ± 0.19 Aa	66 ± 0.17 Aa	66 ± 0.17 Aa
Droplet-Vitrification	72 ± 0.13 Aa	62 ± 0.11 Aa	62 ± 0.11 Aa	60 ± 0.12 Aa
V-Cryoplate	68 ± 0.11 Aa	64 ± 0.09 Aa	64 ± 0.09 Aa	62 ± 0.11 Aa
D-Cryoplate	80 ± 0.10 Aa	78 ± 0.08 Aa	78 ± 0.08 Aa	74 ± 0.05 Aa
Average	72 ± 0.13	68 ± 0.12	67.5 ± 0.11	65.5 ± 0.11

* Data represent the means ± standard error (SE). The same letters are not significantly different according to Tukey’s test (*p* ≤ 0.05). Capital letters indicate comparisons across treatments, within culture time (vertical comparison). Lowercase letters indicate comparisons within treatments across culture times (horizontal comparison).

**Table 2:** Comparison of stem length between in vitro controls and cryo-derived plants of *Vanilla planifolia* during greenhouse culture.

	Greenhouse Culture Time (Months)			
	3	6	9	12
Method		Stem Length (cm ± SE) *		
In vitro control	13.85 ± 3.90 Ac	16.46 ± 4.09 Ac	19.37 ± 4.21 Ab	22.91 ± 4.89 Aa
Droplet-vitrification	14.54 ± 3.15 Ac	16.70 ± 3.17 Ab	18.82 ± 3.16 Aa	20.86 ± 3.06 Aa
V-Cryoplate	14.42 ± 3.18 Ac	16.52 ± 3.42 Ac	18.86 ± 3.17 Ab	21.44 ± 3.26 Aa
D-Cryoplate	14.46 ± 3.73 Ac	16.73 ± 3.96 Abc	19.32 ± 4.40 Aab	22.07 ± 4.70 Aa
Average	14.32 ± 3.49	16.60 ± 3.66	19.09 ± 3.73	21.82 ± 3.98

* Data represent the means ± standard error (SE). The same letters are not significantly different according to Tukey’s test (*p* ≤ 0.05). Capital letters indicate comparisons across treatments, within culture time (vertical comparison). Lowercase letters indicate comparisons within treatments across culture times (horizontal comparison). Initial stem length of acclimatized plants: 12.08 cm on average.

**Table 3:** Comparison of number of leaves between in vitro controls and cryo-derived plants of *Vanilla planifolia* during greenhouse culture.

	Greenhouse Culture Time (Months)			
	3	6	9	12
Method		Number of Leaves (Nr. ± SE) *		
In vitro control	6.53 ± 1.28 Bd	7.60 ± 1.25 Bc	8.90 ± 1.32 Bb	10.27 ± 1.55 Ba
Droplet-Vitrification	6.83 ± 0.95 Bd	8.17 ± 1.02 Bc	9.67 ± 1.12 Bb	11.13 ± 1.28 Ba
V-Cryoplate	8.13 ± 2.00 Ad	9.73 ± 2.07 Ac	11.23 ± 2.22 Ab	12.90 ± 1.92 Aa
D-Cryoplate	7.06 ± 2.02 ABc	8.27 ± 2.12 Bbc	9.67 ± 2.11 Bb	11.10 ± 2.14 Ba
Average	7.13 ± 1.57	8.44 ± 1.61	9.87 ± 1.70	11.35 ± 1.72

* Data represent the means ± standard error (SE). The same letters are not significantly different according to Tukey’s test (*p* ≤ 0.05). Capital letters indicate comparisons across treatments, within culture time (vertical comparison). Lowercase letters indicate comparisons within treatments across culture times (horizontal comparison). Initial number of leaves of acclimatized plants: 5.88 on average.

**Table 4:** Analysis of electrophoretic profiles with the ISSR primers (T05, T06, C07, UBC-823, UBC-836, UBC-840 and UBC-848) to detect polymorphisms in greenhouse-grown *V. planifolia* in vitro controls and in cryo-derived regenerants using three vitrification-based techniques.

ISSR Primer	Amplicons	Total Amplified Bands	No. Polymorphic Bands	Technique	Polymorphism (%)	General Polymorphism (%)	Size (bp)
T05	52	13	0	In vitro Ctrl.	0	0	1200–2800
				D-V	0		
				D-Cp	0		
				V-Cp	0		
T06	30	8	2	In vitro Ctrl.	25	25	730–4000
				D-V	25		
				D-Cp	25		
				V-Cp	33.33		
C07	45	15	9	In vitro Ctrl.	75	60	470–3000
				D-V	75		
				D-Cp	75		
				V-Cp	100		
UBC-823	31	9	3	In vitro Ctrl.	33.33	33.33	1055–3500
				D-V	37.50		
				D-Cp	37.50		
				V-Cp	50		
UBC-836	56	14	0	In vitro Ctrl.	0	0	320–2240
				D-V	0		
				D-Cp	0		
				V-Cp	0		
UBC-840	47	15	7	In vitro Ctrl.	63.63	46.67	920–2500
				D-V	58.33		
				D-Cp	58.33		
				V-Cp	58.33		
UBC-848	25	7	1	In vitro Ctrl.	14.30	14.29	840–1950
				D-V	16.70		
				D-Cp	16.70		
				V-Cp	16.70		
Total	286	81	22			27	

Cryogenic techniques: D-V: droplet-vitrification, V-Cp: V-cryoplate, D-Cp: D-cryoplate. Ctrl: control.

**Table 5:** ISSR primers used for genetic integrity assessment of *V. planifolia* regenerants from cryo-procedures and in vitro-derived plants.

Primer	Annealing Temperature	Sequence (5′–3′)
T05	54 °C	5′ CGTTGTGTGTGTTGTTGT 3′
T06	50 °C	5′ AGAGAGAGAGAGAGAGT 3′
C07	56 °C	5′ GAGAGAGAGAGAGAGAC 3′
UBC-823	49 °C	5′ TCTCTCTCTCTCTCTCC 3′
UBC-836	50 °C	5′ AGAGAGAGAGAGAGAGTA 3′
UBC-840	50 °C	5′ GAGAGAGAGAGAGAGAT 3′
UBC-848	51 °C	5 ′ CACACACACACACACARG 3′

## Data Availability

All data in this study are available in the manuscript.
